# Bridging Gaps in Aquatic Remote Sensing Reflectance Validation: Pixel Boundary Effect and Its Induced Errors

**DOI:** 10.3390/s25237333

**Published:** 2025-12-02

**Authors:** Shuling Xiao, Chunguang Lyu, Chi Zhang, Jochem Verrelst, Ling Wang, Yunfei Shi, Yanmei Lyu, Haochuan Shi

**Affiliations:** 1 College of Resources and Environment, Linyi University, Linyi 276000, China; 2 State Key Laboratory of Desert and Oasis Ecology, Xinjiang Institute of Ecology and Geography, Chinese Academy of Sciences, Urumqi 830011, China; 3 Image Processing Laboratory (IPL), University of Valencia, 46980 Valencia, Spain; 4 Key Laboratory of Radiometric Calibration and Validation for Environmental Satellites, China Meteorological Administration, Beijing 100081, China

**Keywords:** ocean color, pixel boundary effect, pixel-level spatial mismatch index, remote sensing reflectance, uncertainty analysis

## Abstract

Ocean color remote sensing is important for monitoring marine biogeochemical processes. The accuracy of remote sensing reflectance (R_rs_), a fundamental data product, is critical yet challenged by the scale mismatch between in situ point measurements and satellite-based areal observations from pixels. This mismatch introduces uncertainty, notably from the non-uniform spatial response within a pixel—a potential error source at pixel boundaries that remains poorly quantified. To address this issue, we introduced the pixel-level spatial mismatch index (PSMI) to assess spatial representativeness errors induced by the pixel boundary effect (PBE). Using AERONET-OC (AErosol RObotic NETwork-Ocean Color) data alongside MODIS/Aqua and OLCI/Sentinel-3A observations, we showed that the PSMI effectively identified a systematic R_rs_ deviation peak when a site lay within a pixel’s edge attenuation zone. This phenomenon, observed across sensors with different resolutions (MODIS and OLCI), exhibited sensor- and band-dependent peak characteristics. We further proposed a quantitative framework called a Riemann Stieltjes integral-based index to measure the spatial concentration of this deviation peak, and a baseline method to objectively define the PBE window. Our analyses revealed that PBE not only acts as an independent error source but also interacts with atmospheric and geometric errors, forming new multifactor interactions that significantly modulate the overall uncertainty in R_rs_ products. Consequently, pixel-scale effects should be incorporated into future validation protocols, and the PSMI framework can provide an intrinsic tool for this purpose.

## 1. Introduction

Ocean color remote sensing using satellite data serves as a powerful tool for monitoring marine biogeochemical processes on regional to global scales [1,2]. Within this framework, remote sensing reflectance (R_rs_) is a primary data product used to derive key parameters, such as chlorophyll-a concentration [3,4,5]. The confidence in these derived products is closely linked to the accuracy of R_rs_, which is typically evaluated through comparison with high-quality, in situ measurements from networks such as AERONET-OC [6,7,8,9]. However, the inherent scale mismatch between point-based ground measurements and area-averaged satellite pixel observations remains a fundamental challenge in this validation process [10,11]. This scale discrepancy can introduce uncertainties, thereby reducing the overall reliability of satellite-derived biogeochemical products.

Extensive research has been conducted on the validation of reflectance products in aquatic remote sensing and the quantification of their uncertainties. Existing research can be categorized into three directions: (i) refinement of atmospheric correction algorithms, where methods based on shortwave infrared bands, neural networks, or multiband simultaneous inversion have been developed to improve retrieval accuracy in complex coastal waters while addressing the limitations of traditional near-infrared algorithms [12,13,14,15]; (ii) cross-validation of multi-sensor products and uncertainty attribution, with systematic evaluations of sensors such as MODIS, OLCI, VIIRS, and GOCI revealing the sensitivity of their accuracy to various factors (e.g., aerosol type, sun-viewing geometry, water optical properties, and calibration differences) [16,17]; (iii) optimization of validation methodologies themselves, including the development of rigorous uncertainty propagation models based on fiducial reference measurements, and investigations into the effects of spatiotemporal matching windows and viewing geometry corrections on matchup analyses [18,19,20,21].

Despite noteworthy advances in addressing algorithmic and systematic biases, the impact mechanisms and quantification approaches for pixel-scale spatial representativeness errors—inherently arising from the point-to-area scale mismatch—require further in-depth investigation [9,22,23]. Current validation practices often assume that R_rs_ within a satellite pixel is homogeneous and that a ground-site measurement can represent the entire pixel [24,25]. However, the satellite-observed value is a weighted integral of the surface response within the instantaneous field of view, with the weighting following a nonuniform distribution (e.g., approximately Gaussian) [26,27]. This weighting is formally described by the sensor’s point spread function (PSF), and the discrepancy it introduces is called the Pixel Boundary Effect (PBE) in this study. The theoretical foundation for attributing the R_rs_ discrepancy to PBE rests on this intrinsic scale mismatch and spatial response. When an in situ site is located in the pixel’s attenuation zone (where the PSF weighting decays), its contribution to the integrated signal is diminished [28,29]. This leads to a systematic, geometry-dependent bias that manifests as the observed deviation peak. This fundamental scale mismatch between the “point” (ground measurement) and the “area” (pixel-integrated value) introduces errors owing to spatial heterogeneity within the pixel. The issue becomes particularly pronounced when the ground site is located near the pixel boundary, i.e., the region of spatial response decay [30,31,32]. Spatial representativeness has been assessed by incorporating external high-resolution imagery; however, these approaches can introduce additional uncertainties and involve complex procedures. Consequently, developing an intrinsic method, independent of external data and capable of quantifying errors induced by nonuniform pixel spatial response directly from the satellite products themselves, remains a significant gap in the current literature and can help refine the aquatic remote sensing validation framework.

To address this gap, we aimed to extend the Pixel-level Spatial Mismatch Index (PSMI) methodology previously developed for the validation of satellite atmospheric products to the validation of R_rs_ in ocean color remote sensing [33]. This method is based on the construction of a comprehensive metric that integrates pixel geometry (semi-major and semi-minor axes), relative distance, and azimuth information. This metric aims to precisely characterize the positional relationship between the ground site and the pixel’s spatial response field, modeled as an approximate ellipse, thereby providing an intrinsic perspective for understanding the PBE as an independent error source.

Within the PSMI framework, we systematically analyzed the spatial distribution patterns of the relative deviations (rd) of R_rs_ using ground-based observations and multi-sensor satellite data. We further introduced quantitative indices by constructing rd vs. PSMI response curves across different spectral bands and identifying their spatial response characteristics to characterize the concentration of deviation peaks, and described a “PBE window” to identify samples considerably affected by boundary effects. Building on this, we preliminarily investigated the interaction between the PBE and traditional error sources (e.g., atmospheric conditions and sun glint) to enable a more comprehensive assessment of uncertainty contributors in R_rs_ products. This work provides a complementary analytical dimension for validating aquatic R_rs_ products, deepens the understanding of error mechanisms at the pixel scale, and offers insights for the development of future validation protocols and uncertainty control of ocean color satellite products.

## 2. Data and Study Area

### 2.1. AERONET-OC Sites and Study Area

The ground-based station data were obtained from AERONET-OC (aeronet.gsfc.nasa.gov/ (accessed on 13 October 2025).) [34]. This network utilizes sun photometers deployed at more than 40 sites worldwide, providing various water-leaving radiance-related variables at multiple times throughout the day across the visible to near-infrared spectra [6,35]. Among them, the normalized water-leaving radiance derived from inherent optical properties (nLw_IOP) is a normalized product derived from inherent optical property modeling, offering relatively high data reliability [36,37,38].

Considering factors such as cloud screening and quality control, we selected Level 2.0 nLw_IOP data from the following three sites (see Table 1) for the period 2016–2023, which provides an extensive temporal coverage. These platforms are located in waters with minimal terrestrial disturbance, typically under oligotrophic to mesotrophic conditions, exhibiting better data continuity and quality [39,40]. Their use as a benchmark dataset is advantageous for conducting synchronous matchup comparisons with relevant satellite-derived products.

### 2.2. Satellite Data

**Figure 1:**
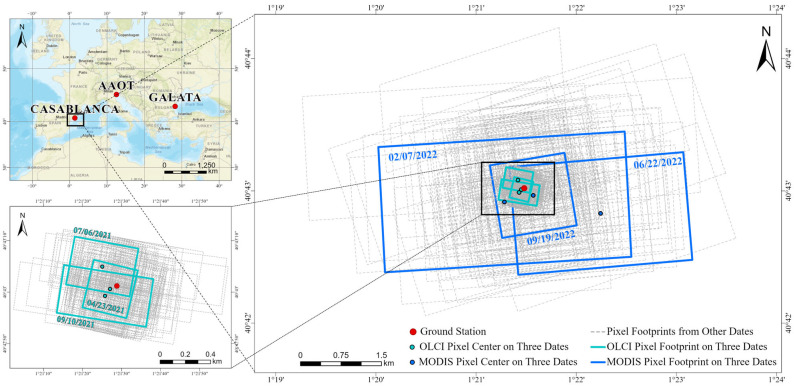
Location of the study area and pixel spatial coverage. The spatial distribution of all sites is shown at upper left, with the Casablanca platform’s adjacent OLCI and MODIS pixels detailed at lower left and right, respectively. Data from three representative dates are highlighted in bold, colored solid lines to clarify the pixel-site relationship (blue for MODIS, green for OLCI).

We primarily utilized two representative satellite-derived water-leaving radiance products: the MODIS/Aqua Level-2 regional ocean color data (MODISA_L2_OC) and the OLCI/Sentinel-3A Level-2 full resolution earth observation product (OLCIS3A_L2_EFR_OC). The MODIS and OLCI sensors are aboard NASA’s Aqua and ESA’s Sentinel-3A satellites, respectively [41,42]. Both products provide data in the form of normalized R_rs_. The spatial resolutions of the MODISA_L2_OC and OLCIS3A_L2_EFR_OC products are 1 km and 300 m, respectively. They consistently employ the multiband atmospheric correction algorithm provided by the Ocean Color website (oceancolor.gsfc.nasa.gov/ (accessed on 13 October 2025).) [43]. Bands adjacent to 412, 443, 490, and 667 nm were selected from the aforementioned satellite products, with the central wavelength deviation not exceeding 2 nm, to match the spectral channels of the AERONET-OC data within the same spatiotemporal domain (Figure 1).

## 3. Methods

**Figure 2:**
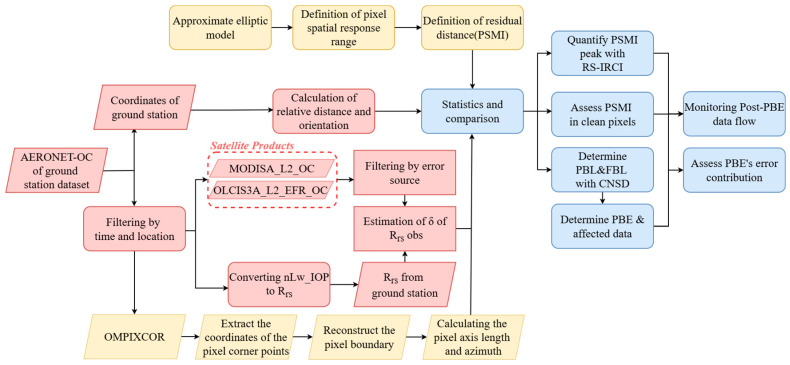
Data and workflow for evaluating the influence of PBE. It outlines the methodology, which includes data preprocessing (in red), pixel reconstruction and indicator derivation (in yellow), and results assessment (in blue), ultimately to define pixel geometry and quantify boundary-driven errors.

As illustrated in Figure 2, the methodology for assessing the degree of PBE influence is structured into three main phases: (1) Data preprocessing (in red), involving spatiotemporal filtering and estimation of the relative deviation (δ) between ground-based and satellite-derived R_rs_; (2) Pixel reconstruction and indicators (in yellow), which utilizes the OMPIXCOR dataset to determine pixel spatial boundaries, calculate pixel axis length, and develop a novel pixel-level spatial metric; (3) Results assessment (in blue), comprising various evaluations of δ using specific spatial indicators, thereby quantifying the contribution of PBE within the error sources.

### 3.1. Data Preprocessing

#### 3.1.1. Radiometric Harmonization

The conversion between the normalized water-leaving radiance (nLw, with units of W·m^−2^·sr^−1^·μm^−1^) measured by AERONET-OC sensors and the satellite-derived R_rs_ (with units of sr^−1^) product is defined by the following relationship [44,45]:

(1) $$R_{rs}=nLw/F_{0,}$$

where *F*_0_ represents the mean extraterrestrial solar irradiance at the top of the atmosphere. In this study, the nLw_IOP data were converted to R_rs_ using the Thuillier dataset for *F*_0_, as recommended by the Ocean Biology Processing Group [46].

#### 3.1.2. Spatiotemporal Colocation Criteria

A spatiotemporal collocation was performed to filter the matchup analysis samples and accurately capture the relationship between the satellite-ground observation geometry and the observation bias of R_rs_ within the study area. Based on the established practices for validating satellite R_rs_ products, the maximum allowed time difference between the AERONET-OC and satellite observation was set to three hours, which ensured a sufficient number of observation samples for statistical analysis [47]. For each satellite overpass, the three pixels closest to the AERONET-OC site were identified. The R_rs_ values of these pixels were then used to assess spatial variability. This approach provides multiple observations per satellite passage for a more robust statistical analysis.

#### 3.1.3. rd Calculation

For each successfully matched sample after spatiotemporal collocation, the rd (δ) between the satellite and ground-based R_rs_ was calculated using the following formula:

(2) $$\delta_{i}=\left|\frac{{Rrs}_{s,i}-{Rrs}_{g,i}}{{Rrs}_{g,i}}\right|\times 100\%,$$

where Rrs_s,i_ and Rrs_g,i_ are the R_rs_ values from the satellite and the AERONET-OC ground station for the *i*-th matchup, respectively. The AERONET-OC measurement (Rrs_g,i_) was used as the reference value.

#### 3.1.4. Error Source Identification

The processing flags embedded within the MODIS and OLCI Level-2 ocean color products are listed in Table 2 [8,48]. The error sources flagged at the pixel level can be categorized into several types, including Atmosphere and Surface, Observation, Sensors, and Model and Algorithm. These error sources impact the satellite observations both independently and interactively.

Notably, the native flags such as LOWLW and HISOLZEN only identify a subset of extreme conditions. Therefore, in this study, we implemented additional identification and flagging criteria for cases where the water-leaving radiance fell below 1 × 10^−5^ W m^−2^ sr^−1^ μm^−1^ and where the solar zenith angle exceeded 60°.

### 3.2. Pixel Spatial Reconstruction and Parameterization

#### 3.2.1. Pixel Spatial Reconstruction

Figure 1 shows the spatial coverage of representative MODIS and OLCI pixels. Based on the existing metric algorithm, the Vertex of the Pixel (VP) was determined using the latitude and longitude of each filtered pixel and its four adjacent pixels in the dataset [33,49,50,51]. Starting from the Center of the Pixel (CP), trajectories were generated along the across-track and along-track directions by connecting to the CPs of the adjacent pixels. These trajectories intersected the pixel boundary at the Endpoints of the Pixel Axis (EPA), thereby defining the left and right segments in the across-track direction and the upper and lower segments in the along-track direction, as divided by the CP. The latitude and longitude of each EPA and VP were calculated using the equipartition-intersection method [33]. Specifically, an EPA reflected the midpoint of the axis between the primary pixel and a neighboring pixel in each of the four directions. A VP was then determined by extending the axes from two adjacent EPAs and finding their intersection point, ultimately defining the pixel’s boundary. For a detailed schematic representation of the geometric relationships between these variables, refer to the Supplementary Section S1.

#### 3.2.2. Parameterization

To reveal the connection between the satellite-ground observation geometry and rd, the defined PSMI is expressed as follows [33]:

(3) $$PSMI=d-L_{\theta ,}$$

where *d* is the distance between the pixel center and the ground station, calculated as:

(4) $$d=re\cdot arccos\left(\varphi \right).$$

Here, *re* is the Earth’s radius at the station’s location, and *φ* is the central angle, which can be obtained from:

(5) $$\varphi =\cos\left(\frac{\pi }{2}-y\right)\cdot cos\left(\frac{\pi }{2}-Y\right)+sin\left(y\right)\cdot sin\left(Y\right)\cdot cos\left(x-X\right),$$

where *x*, *y* and *X*, *Y* are the longitude and latitude of the ground site and the satellite pixel center, respectively. *L*_θ_ is the distance from the pixel center to the corresponding ellipse with the rotation angle θ, given by:

(6) $$L_{\theta }=\sqrt{{\left\{a\cdot cos\left[\alpha \left(\theta \right)\right]\right\}}^{2}+{\left\{b\cdot sin\left[\alpha \left(\theta \right)\right]\right\}}^{2}},$$

Here, *a* and *b* are the semi-major and semi-minor axes of the ellipse determined for the corresponding angular direction, respectively. *α* is the eccentric angle corresponding to the rotation angle *θ*, and their relationship is described by:

(7) $$\alpha \left(\theta \right)=arctan\left[\frac{a}{b}\cdot tan\left(\theta \right)\right],$$

The symbols in the equation are defined as above. The PSMI represents the residual between L_θ_ and d and its magnitude reflects the distance metric that accounts for the pixel’s spatial response. A negative (positive) PSMI value indicates that the ground station is located inside (outside) the spatial response ellipse.

### 3.3. Assessment of Results

#### 3.3.1. PSMI Deviation Peak Morphology

When the rd of R_rs_ across spectral bands is influenced by significant pixel spatial response variations, the PSMI profile exhibits a multi-peak morphology centered around a dominant peak (the maximum rd value). Along the PSMI dimension (*x*-axis), the overall sample distribution approximates a bell-shaped structure with varying amplitudes of rd. Conversely, when viewed along the rd dimension (*y*-axis) starting from the dominant peak, the sample set can be transformed into a range set. The concentration degree of the PSMI deviation peak at the dominant peak position is quantified using a Riemann Stieltjes integral form [52,53]:

(8) $$\eta =\int_{0}^{1}PxdPy,$$

where *η* is the Riemann Stieltjes Integrated Range Concentration Index (RS-IRCI), *Px* describes the proportion of the spatial range required to reach a specific cumulative amplitude level, and *Py* is the cumulative probability scale (from 0 to 1). In practical computation, *η* can be approximated discretely as

(9) $$\eta =\sum_{k=2}^{N}Px\left(k\right)\Delta Py\left(k\right),$$

where N is the total number of data points. *Px*(k) and *Py*(k) can be regarded as the cumulative range ratio functions in the X and Y directions, respectively. For the top k samples with the largest amplitudes in the set, they are defined as

(10) $$\left\{\begin{array}{c} Px\left(k\right)=R_{x}\left(k\right)/R_{x}\\ Py\left(k\right)=R_{y}\left(k\right)/R_{y} \end{array}\right..$$

Here, *R_x_* and *R_y_* are the global ranges in the *X* and *Y* directions, respectively, whereas *R_x_*(*k*) and *R_y_*(*k*) are the local ranges for the top *k* samples in their respective directions. Consequently, in Equation (9), Δ*Py* can be viewed as a nonuniform weight measure, expressed as

(11) $$\Delta Py\left(k\right)=Py\left(k\right)-Py\left(k-1\right).$$

In summary, the RS-IRCI can be concretely interpreted as the area of the minimum bounding rectangle encompassing the sample set in the *Px Py* space. The RS-IRCI serves as a normalized metric enabling cross comparison between PSMI profiles from different data sources, as illustrated in Supplementary Section S3 Figure S3, Tables S1–S3 [50,54,55,56,57,58,59,60,61]. Its value provides an intuitive measure of the typicality of the PSMI deviation peak.

#### 3.3.2. PBE Window

The RS-IRCI, built upon the sample range, effectively characterizes the overall structure of the PSMI profile. However, it is necessary to incorporate metrics that reflect the dispersion of the samples, such as the standard deviation, into a comprehensive model to delineate the internal heterogeneities within the PSMI profile and investigate the effective interval of the PBE.

By referring to the normalization method for *Px*(*k*) and *Py*(*k*), we obtain

(12) $$\zeta \left(k\right)=\sigma_{y}\left(k\right)/\sigma_{y},$$

where *ζ* is the Cumulative Normalized Standard Deviation (CNSD), *σ*_y_(*k*) is the local standard deviation of the top k samples with the largest amplitudes in the δ dimension, and σ_y_ is the global standard deviation of the entire sample set. Similar to *Px*, *ζ* generally exhibits an increasing trend from 0 to 1 over the *Py* range. The CNSD is sensitive to the distribution of sample points along the PSMI interval and can be used to identify the baseline of the PSMI deviation peak.

Based on this, a weighting coefficient *ω*(*k*) is defined to detect the positions of abrupt numerical changes as a function of *Py*, as follows:

(13) $$\omega \left(k\right)=∆\zeta \left(k\right)\cdot Py\left(k\right).$$

Here, Δ*ζ*(*k*) is the increment at the *k*-th sample (when sorted by the largest amplitude), and *Py*(*k*) serves as a weight value. Consequently, a set of baselines can be derived through the transformation in Equation (14):

(14) $$\left.\begin{array}{c} \begin{array}{c} \zeta \left(k\right)\in R\to 1 \end{array}\\ \zeta \left(k\right)\notin R,Py>0.5 \end{array}\right\}⟹max\left[\omega \left(k\right)\right]⟹\left\{\begin{array}{c} PBL\\ FBL \end{array}\right..$$

Here, the Primary Base Line (PBL) and Functional Base Line (FBL) are obtained from the δ values at the positions (k) where ω(k) reaches its maximum within two specific value ranges of ζ(k), respectively. Together, they indicate the transition intervals in the distribution state of the PSMI samples. Physically, the PBL and FBL identify the effective interval of the pixel boundary effect within the PSMI profile, with the PBL marking its onset and the FBL characterizing its fully developed state.

The PBL marks the demarcation point where *σ*_y_(*k*) approaches and reaches the overall dispersion level of the PSMI samples, i.e., where *ζ*(*k*) becomes saturated (in this study, *ζ*(*k*) ranged from 0.9 to 1.2). The FBL is defined as the largest critical value within the lower half of the PSMI deviation peak (*Py* > 0.5) during the evolution of *ζ*(*k*) toward its saturated state.

As shown in Supplementary Section S3 Figure S3d, a “compact” sample distribution is evident within the PBL. The FBL, in contrast, identifies more of the minor fluctuations (“noisy” samples) within the PSMI threshold range, possessing the ability to maximize the separation of samples unrelated to the pixel’s spatial boundary. This is also the reason it is named the FBL.

Naturally, based on this, the PBE window (**W**) is described by

(15) $$W=R_{PSMI}\left(k\right),rd\left(k\right)>FBL.$$

Here, *R_PSMI_*(k) represents the local range in the PSMI dimension for the top *k* samples with the largest δ values, and the range of *k* is determined by the FBL. When PSMI sample points fall within the window **W**, they maintain or even enhance the morphology of the PSMI deviation peak, regardless of their specific location. Conversely, PSMI sample points located outside both **W** and the global *R_PSMI_* range tend to distort the morphology of the PSMI deviation peak.

#### 3.3.3. Error Source Correction

First, a PSMI profile was constructed using clean pixel samples unaffected by any error sources (see Section 3.1.4). The window construction algorithm from Section 3.3.2 is then applied to identify the subsets within both the clean samples and the error source-affected samples that were significantly influenced by the PBE. Subsequently, the clean samples identified as being influenced were labeled as the PBE. The error source-affected samples were recursively categorized under the corresponding multifactor interaction cases. This process completes the quantification of error source contributions—including the PBE—to the rd of water-leaving R_rs_ across various spectral bands.

## 4. Results

### 4.1. Correlation and rd

**Figure 3:**
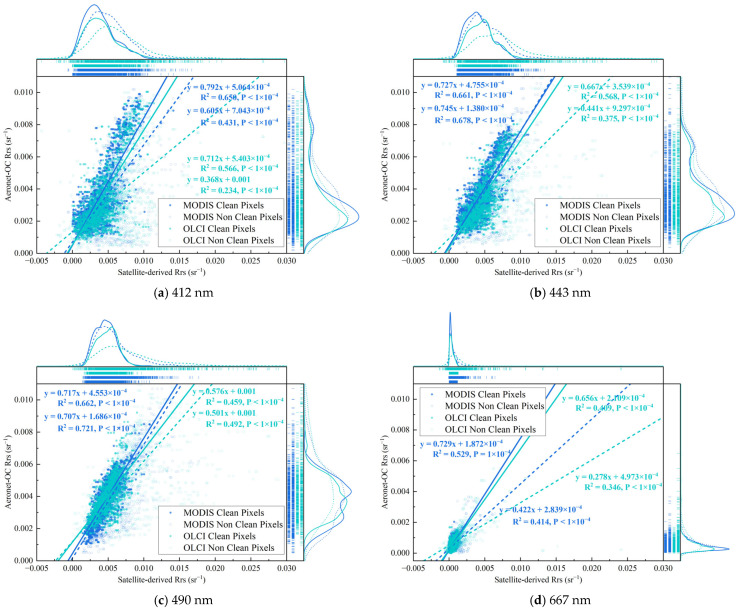
Correlation between satellite and ground-based R_rs_ data. This scatter plot compares satellite-derived products against AERONET-OC reference data. Data points from MODIS and OLCI are color-coded in blue and green, respectively. Open circles denote non-clean pixels, while filled circles represent clean pixels. Solid and dashed lines correspond to clean and non-clean pixels, respectively. Each data group is fitted with a regression line, whose equation is provided alongside the R^2^ and *p*-value to quantify the agreement.

Figure 3 shows the correlation between satellite-derived (MODIS and OLCI) and ground-based AERONET-OC R_rs_ data. For the detection bands at 412, 443, and 490 nm, the clean pixel R_rs_ samples exhibited reasonably good correlations (MODIS: R^2^ > 0.6, *p*-value << 0.05; OLCI: R^2^ > 0.4, *p*-value << 0.05). Furthermore, the correlation for the MODIS product was generally higher than that for the OLCI product across these bands. MODIS and OLCI achieved their highest R^2^ values at 490 nm (0.662) and 443 nm (0.568), respectively. In contrast, the 667 nm band showed a notably smaller value range and a lower level of correlation, owing to its weak aquatic signal where a low signal-to-noise ratio allows uncorrelated noise to obscure the true linear relationship. Following the application of the clean pixel screening, the data correlation was generally enhanced across the spectrum. Notably, at the 443 and 490 nm bands, the correlation coefficients for clean pixels approached or, in some instances, were comparable to those of non-clean pixels. Despite this, non-clean pixels consistently exhibited a higher degree of scatter overall, indicating the presence of substantial observational deviations introduced by various error sources.

**Figure 4:**
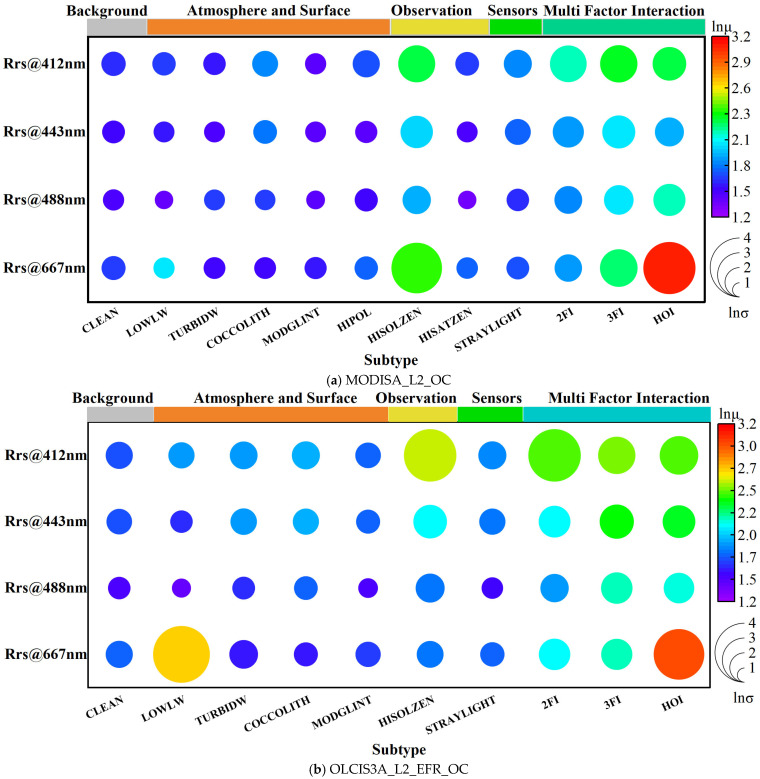
Statistical characteristics of the rd of R_rs_ across wavelengths and error sources. The color bars at the top denote different categories, with the final one, “Multi Factor Interaction”, corresponding to the interaction terms detailed in Table 2. The circle size and color represent the natural logarithm of the variance and the mean, respectively. Thus, larger, redder circles indicate higher values.

Figure 4 presents the statistical characteristics of the rd of R_rs_ in logarithmic form for each wavelength and error source. Apart from the clean pixels defined as the “Background” type, the error sources in the current sample sets of the MODIS and OLCI products (described in Table 2) altered the mean and standard deviation of the rd of R_rs_ by acting either independently or interactively. The interactive effects were further subdivided into Two-Factor Interactions (2FIs), Three-Factor Interactions (3FIs), and Higher-Order Interactions (HOIs, representing interactions of four or more error sources) based on the number of contributing error sources.

Independent factors such as LOWLW, TURBIDW, COCCOLITH, MODGLINT, and HIPOL exhibited a minor impact at the 412, 443, and 490 nm band. In contrast, interactive effects involving multiple factors, particularly the HOI, significantly amplified both the mean and standard deviation of the rd. For the 667 nm band, besides its background rd being higher than those in other bands, it showed a strong response to both single-factor and multifactor influences (in Figure 3d). Additionally, HISOLZEN played a significant role as an independent error source in both sensors, while LOWLW exhibited a more pronounced influence, particularly in the OLCI product, reflecting certain differences in the observational capabilities of the two sensors.

### 4.2. Shape of the PSMI Deviation Peak

**Figure 5:**
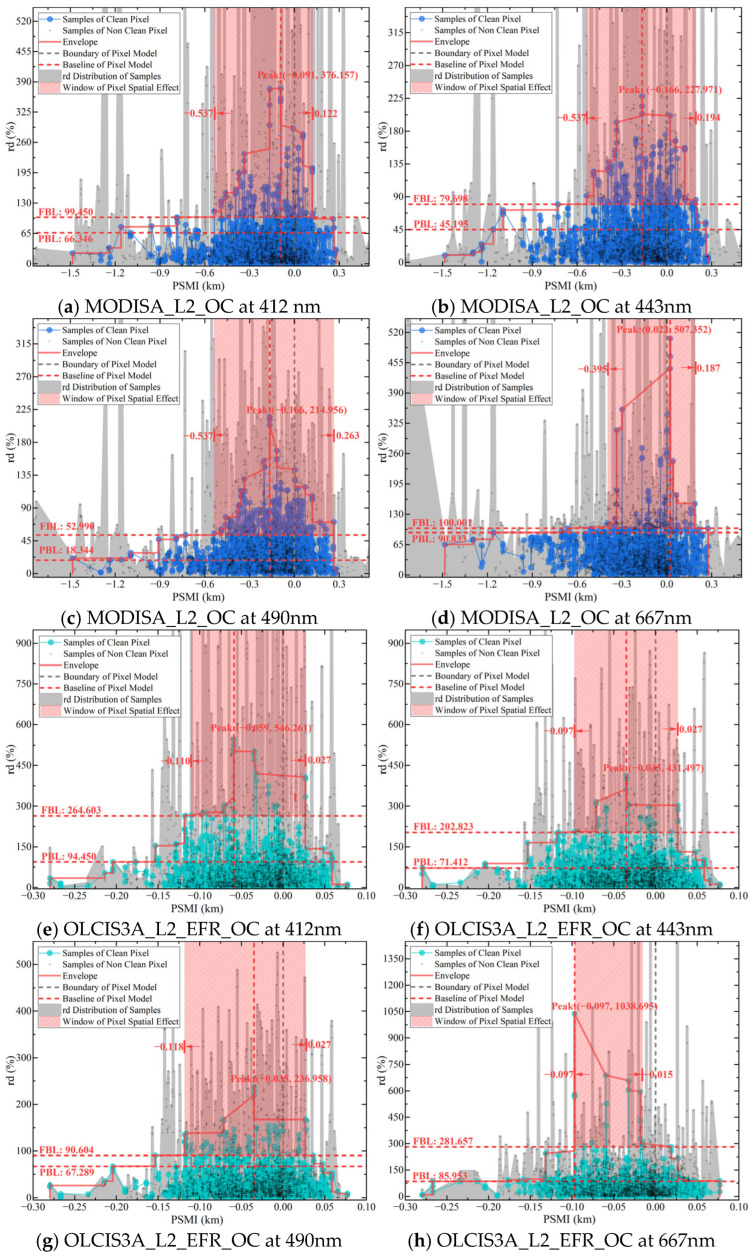
The PSMI peak curve, baselines, and PBE window. The gray shaded area denotes the data series including non-clean pixels, while the colored dots and the colored solid lines represent the series with clean pixels only. The concentration of the rd error peak, delineated by the red envelope, is quantified using values from Figure 6. Furthermore, the Functional Baseline, Primary Baseline, and the Window of Pixel Spatial Effect are determined based on the criteria established in Figure 7.

Figure 5 displays the PSMI profiles constructed from the clean-pixel R_rs_ samples of MODIS and OLCI. The MODIS samples were distributed along the PSMI axis (from −1.480 to 0.277), with the rd peak for each band located between −0.166 and 0.022. Correspondingly, the OLCI product’s PSMI distribution spanned from −0.280 to 0.077, with its peak positions ranging from −0.097 to −0.035. Judging from the peak distribution interval, we deduced that the PSMI rd peaks for OLCI were located closer to the actual pixel boundary.

In terms of peak magnitude, the OLCI samples at 412, 443, and 490 nm exhibited rd peak values of 546.261, 431.497, and 236.958, respectively, which are significantly higher than those of the MODIS samples at the corresponding wavelengths. However, both sensors exhibited considerably higher peak values at 667 nm compared to the other three bands. These peak morphology observations are consistent with the correlation and rd findings presented in Section 4.1.

**Figure 6:**
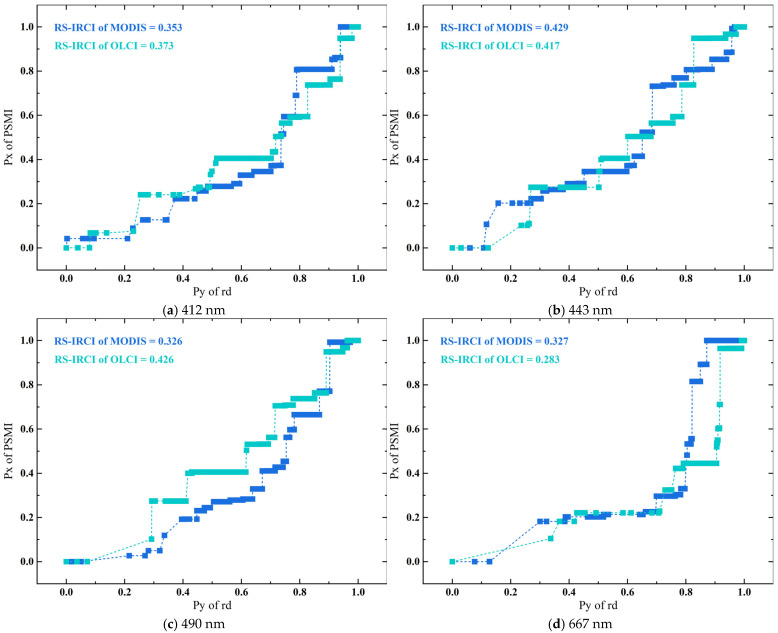
Energy level diagram of the cumulative range ratio function and RS-IRCI. The RS-IRCI quantifies the spatial concentration of rd error peaks, where a smaller value indicates a more concentrated peak distribution and a more pronounced pixel spatial response.

**Figure 7:**
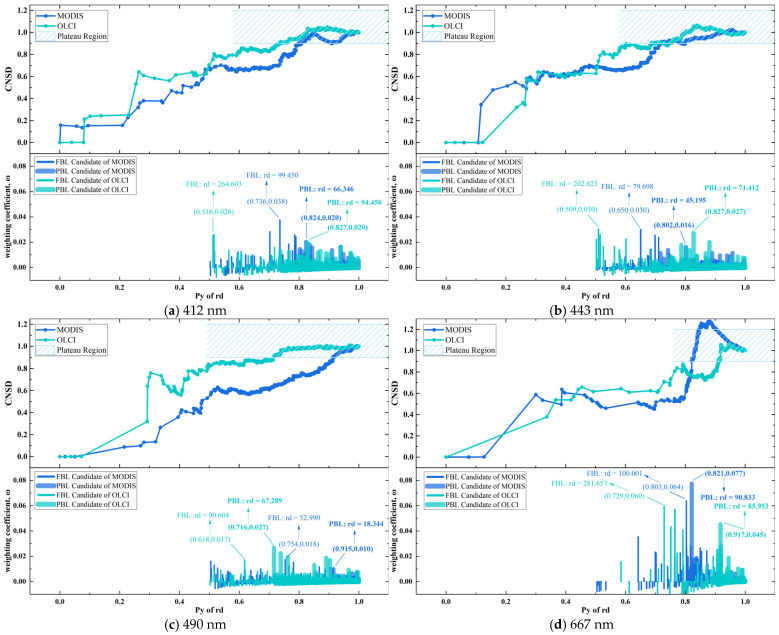
Cumulative normalized standard deviation and the corresponding weighting coefficient. The FBL is identified as the final ascending breakpoint preceding the plateau region, while the PBL is the first such breakpoint within it. Based on the FBL, the Window of Pixel Spatial Effect is then determined, as shown in Figure 5.

Moreover, Figure 5 shows the envelope reflecting the PSMI peak concentration, baselines such as PBL and FBL that characterize the sample distribution, and the window demarcating the PBE. Specifically, the envelope range, derived using the method described in Section 3.3.1, can be transformed into cumulative range ratio functions. This transformation allows for the calculation of the key metric, RS-IRCI, visualized in the energy-level diagram in Figure 6. The extraction of the PBL and FBL (see Figure 7) enables the determination of the PBE window by identifying the “flat” intervals in the rd range. Currently, the PBE window establishes the benchmark for quantifying the PBE. Figure 6 shows the use of RS-IRCI values to assess the concentration degree of the PSMIRD peaks for the two satellite products. The curves represent the cumulative range ratio functions, which exhibit normalized results and a monotonically increasing transition form. The area under the curve relative to the *x*-axis is the RS-IRCI. A smaller RS-IRCI value indicates a more concentrated deviation peak and a more significant pixel spatial response. The data showed that the RS-IRCI ranges for MODISA_L2_OC and OLCIS3A_L2_EFR_OC were 0.326–0.429 and 0.283–0.426, respectively, both below 0.5; thus, these values fall within an acceptable concentration interval according to the definition of the RS-IRCI and in comparison with Figure S2. Overall, the RS-IRCI indicated considerable aggregation effects around the dominant rd peak in the PSMI profiles of both satellite products. However, the deviation peaks for MODISA_L2_OC were more concentrated, suggesting a more pronounced pixel spatial response.

Figure 7 shows CNSD variations across the Py range from 0 to 1 for each band, along with the resulting pulse characteristics of the weighting coefficient ω. As a cumulative metric, the CNSD started at 0, increased with fluctuations, saturated near 1, and subsequently exhibited an oscillatory trend. The CNSD meticulously characterizes how closely the dispersion of all interim states of the PSMI profile—from the point of maximum peak until encompassing all sample points—approaches that of the complete profile. Correspondingly, ω identifies the position and amplitude of mutation points in the trend of the CNSD curve (displayed as pulses in the figure). Following the method in Section 3.3.2, the optimal positions for the PBL and FBL were inferred by identifying the pulse maximum transition points across the two phases defined by whether the CNSD has reached saturation.

The maximum pulse amplitude of ω varied depending on the sensor and detection band. At 667 nm, both products’ samples achieved significantly high values outside the saturation interval, thereby delineating a stable baseline “terrace” for the corresponding PSMI rd peaks. For other scenarios, a screening strategy based on relative pulse highs yielded baseline positions consistent with those determined by direct visual inspection of the PSMI rd peak morphology. A clear pattern emerged from the analysis of both sensors: the PBL occurred earlier in OLCI than in MODIS across all bands, with the exception that the FBL appeared later in OLCI at the 667 nm band. In terms of pulse morphology, the waves were relatively subdued in the first three bands, while a pronounced pulse high was observed at the 667 nm band for both products. This pronounced pulse is likely due to the fewer available samples at 667 nm, where the normalization process to a maximum of 1 amplifies the relative contribution from each individual sample compared to the other three bands. Similar features are reflected in the sample distribution trends for the respective sensors shown in Figure 5. In summary, the pulse maximum transition point rule considers the sample distribution dispersion within the PSMI deviation peak and provides an optimal solution for baseline (i.e., PBL and FBL) identification.

### 4.3. Error Source from the PBE

Figure 5 shows the PBL and FBL for the PSMI deviation peaks of MODIS and OLCI across the bands, along with **W** determined jointly by the distribution of the corresponding envelope. As presented in Table 3, the PBL, corresponding to the saturation interval of the CNSD, provided the optimal correlation and accuracy for ground station validation while incorporating as many sample points as possible into a stable baseline terrace. In comparison, the FBL, identified from the unsaturated interval of the CNSD, accommodated more samples with minor fluctuations across the PSMI rd peak distribution range, providing the maximum threshold for delineating intervals of significantly contrasting concentrations across the PSMI samples.

The window **W** defines the significant domain of the influence of the PBE, independent of the overall sample distribution. Within this range, both clean and non-clean samples enhanced the concentration of the PSMI deviation peak, regardless of their position relative to the envelope. According to Figure 5, for non-clean samples (represented by cross marks in the grey distribution areas), **W** captured those significantly affected by the PBE. Outside **W**, the PSMI deviation peak morphology constructed from clean samples did not respond to sample points located inside the envelope. Conversely, samples outside the envelope distorted or even disrupted the existing PSMI deviation peak morphology. In other words, the intensification of such error effects can mask the PBE information characterized by the PSMI.

**Figure 8:**
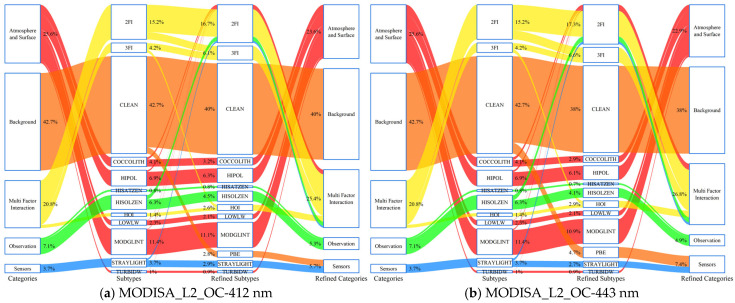

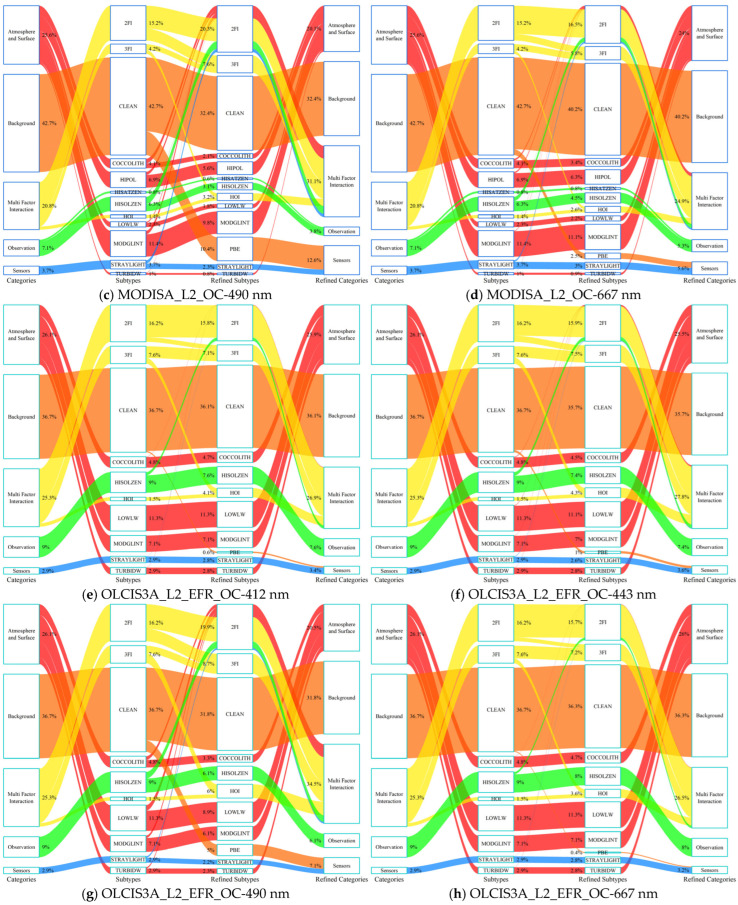
Error source migration induced by PBE assessment. The schematic contrasts data classification before (left two columns) and after (right two columns) introducing PBE. Comparing the values in the outermost columns clarifies the impact of PBE on the data.

Figure 8 presents the error source migration results after introducing the PBE assessment. The two left columns show the original error types and identifiers for the samples, whereas the two right columns show the error types and identifiers after PBE assessment. The proportion of samples induced by the PBE varied with the detection band. For 412, 443, and 490 nm, the proportions for MODIS were 7.4%, 10.7%, and 20.6%, whereas for OLCI, they were 2.2%, 3.5%, and 14.2%, respectively. At 667 nm, the proportion induced by the PBE was minimal for both products, at 6.7% and 1.6%, respectively, which is closely related to the narrower **W** at this band.

In terms of broad categories, the PBE-induced error is closely related to the spatial response mechanism of the satellite sensor pixel and must be classified under Sensors. The combination of pre-existing independent or interactive error sources with the PBE generated new multifactor interaction categories. Consequently, the proportions of these two types notably increased: Sensors (MODIS: ↑1.9–8.9%; OLCI: ↑0.3–4.2%) and Multifactor Interaction (MODIS: ↑4.1–10.3%; OLCI: ↑1.2–9.2%). Correspondingly, the proportions of Background (MODIS: ↓2.5–10.3%; OLCI: ↓0.4–4.9%), Atmosphere and Surface (MODIS: ↓1.6–5.5%; OLCI: ↓0.1–5.6%), and Observation (MODIS: ↓1.8–3.3%; OLCI: ↓1–2.9%) decreased.

**Figure 9:**
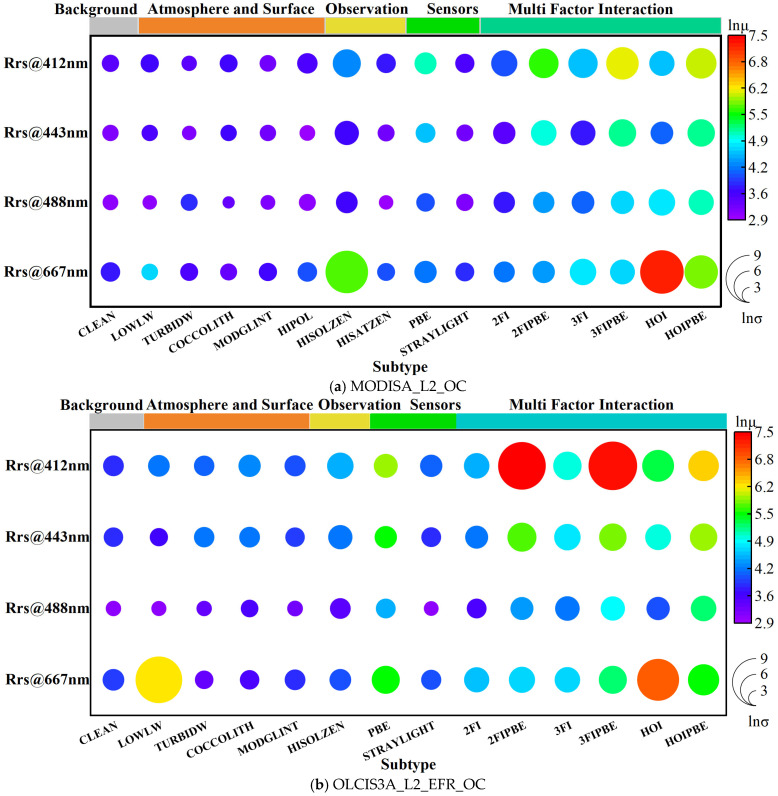
Contributions of error sources to the rd of R_rs_ across the spectrum: The role of the PBE. This picture builds upon Figure 4 by incorporating data influenced by the Pixel Boundary Effect (PBE) as an additional error source. The single-factor categories retain their previous meanings. To isolate the impact of PBE, the multi-factor analysis separately compares datasets with and without PBE influence.

The statistical characteristics of R_rs_ rd for error sources induced by the PBE are shown in Figure 9 using a logarithmic scale and compared with the results shown in Figure 4. Among them, 2FI, 3FI, and HOIs involving the PBE are labeled as 2FI_PBE, 3FI_PBE, and HOI_PBE, respectively. For MODIS, the PBE was the most significant single-factor effect after HISOLZEN across all bands. For OLCI, HISOLZEN and LOWLW remained significant, whereas the PBE at 667 nm was the second most significant factor after LOWLW. In multifactor interactions, the PBE-induced 2FI_PBE and 3FI_PBE generally led to a more pronounced increase in rd compared with the original factor combinations. Notably, at 667 nm, HOI_PBE became the secondary error source after HOI for the MODIS R_rs_ product. For the OLCI product, both 2FI_PBE and 3FI_PBE across all bands were higher than other multi-factor interactions, highlighting differences between the two products. These results show that the PBE, whether as a single factor differentiated from originally clean samples or as a new multifactor type inducing other error sources, exhibits a strong response with the rd of R_rs,_ highlighting the impact of intra-pixel spatial heterogeneity related to the PSMI on the accuracy of R_rs_ products.

## 5. Discussion

### 5.1. PBE: Toward a Revised Interpretation of Validation Anomalies

Our results suggest that the PBE may be a previously underemphasized error source in validation practices in complex aquatic optical environments. This finding offers a new perspective for understanding uncertainties in water R_rs_ products [62,63].

PSMI analysis provides an alternative explanation for some of the high-deviation samples in validation datasets. We observed that in the scatter plots of satellite versus ground-based observations (Figure 3), samples deviating significantly from the 1:1 line exhibited a certain spatial regularity: they showed a high probability of being located within the PBE window (Figure 5), indicating that the observational geometric relationship is a non-negligible covariate. In addition to known atmospheric or water optical factors, the relative positional relationship between the ground site and the pixel’s spatial response field may be an important factor contributing to some of the observed deviations [44,64,65,66]. Consequently, after removing samples significantly affected by the PSMI, the validation correlation across all wavebands showed marked improvement [67,68].

Systematic differences observed in the validation of sensors with different spatial resolutions (e.g., MODIS vs. OLCI) may reflect the modifying effect of the pixel scale on spatial errors. We found that the lower-resolution MODIS (~1 km) exhibited higher correlation with ground observations in the visible band, and its PSMI deviation peak was more concentrated (Figure 6). This observation aligns with studies reporting a systematic underestimation of R_rs_ by OLCI in complex waters [69,70,71]. The higher-resolution OLCI is more sensitive to microheterogeneity within its pixels, thereby exacerbating uncertainties in point area scale matching. In contrast, the larger pixel size of MODIS tends to smooth internal variability, making it statistically more consistent with single-point measurements. Furthermore, MODIS’s longer operational history and more mature algorithm optimization may collectively contribute to its performance, indicating that the pixel scale itself strongly influences validation outcomes when evaluating multisource satellite data.

Substantial interactions exist between the PBE and traditional error sources. Error source migration analyses (Figure 8) revealed that after introducing PBE assessment, some samples originally attributed to atmospheric or observational geometry errors were reclassified as PBE-related. Furthermore, multifactor interaction errors involving the PBE (e.g., 2FI_PBE, HOI_PBE) demonstrated a pronounced amplifying effect on the rd of R_rs_ (Figure 9). This agrees with the established understanding that the coupling of multiple error sources increases the overall uncertainty and suggests that the low-response signal at pixel boundaries might be more susceptible to interference from other factors, thus acting as an “error amplifier.” Therefore, incorporating the PSMI into the uncertainty attribution framework helps to more completely deconstruct the error composition.

Overall, our study reveals the potential modulating role of the PBE, as a form of spatial representativeness error within the complex error system rather than negating the dominant role of traditional error sources, such as atmospheric conditions and water optical properties [72,73,74]. The PSMI provides an intrinsic tool for the systematic identification and quantification of the PBE, opening a supplementary pathway for understanding validation anomalies, optimizing validation strategies, and reducing overall uncertainty.

### 5.2. Regional Features of the PBE

The expanded multi-site analysis robustly confirms that the PBE is a fundamental and ubiquitous phenomenon in aquatic remote sensing validation. However, its specific characteristics, namely the magnitude of the systematic deviation and its spatial concentration, exhibit significant and interpretable regional dependencies across the three AERONET-OC sites (AAOT, Casablanca Platform, Galata Platform).

**Figure 10:**
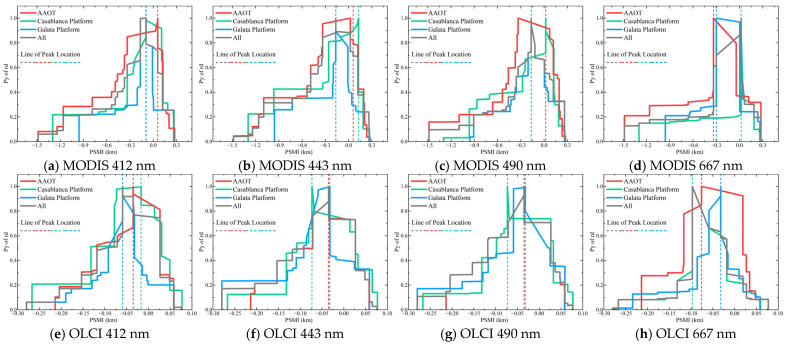
Changes in the PSMI curves in each study area. Envelope curves and peak locations of error peaks across sites. A smaller area enclosed by the envelope and axes indicates a higher spatial concentration of errors (for methodology, see Figure 7).

As synthesized in Table 4, Table 5 and Table 6 and illustrated in Figure 10, distinct rd peaks in R_rs_ consistently emerge near the theoretical pixel boundary across all sites and sensors, though their precise locations exhibit systematic, sensor-dependent patterns. When accounting for the respective native pixel sizes, OLCI (300 m resolution) shows peaks concentrated within a PSMI range of −0.278 to 0.077 km, consistently located inside the pixel (PSMI < 0), with site-specific concentrations between −0.097 and −0.017 km at Casablanca and −0.032 to −0.076 km at the other sites. In contrast, MODIS (1 km resolution) exhibits peaks across a PSMI range of −1.485 to 0.277 km, distributed on both sides of the boundary. This scaling relationship suggests that the PBE manifests proportionally to the native pixel dimensions. The consistent inward shift in OLCI peaks may indicate its heightened sensitivity to sub-pixel heterogeneity due to finer spatial resolution. Beyond sensor characteristics, the local aquatic optical environment could also modulate the PBE’s intensity, as evidenced by the OLCI 412 nm peak rd increasing from 73.4% at the Casablanca platform to 546.3% at the Galata platform, suggesting that sub-pixel heterogeneity in coastal waters amplifies the PBE.

Beyond the peak magnitude, the spatial structure of the PBE, quantified by the RS-IRCI, further reveals regional nuances. A lower RS-IRCI signifies a more spatially concentrated rd peak. Our results show that the rd distribution is typically more concentrated (lower RS-IRCI) at Casablanca and Galata across most bands compared to AAOT. For instance, the RS-IRCI for OLCI at Galata ranges from 0.252 to 0.418, whereas at AAOT, it spans from 0.289 to a notably higher 0.611, particularly in the 667 nm band. This indicates that at AAOT, high-deviation samples are distributed across a broader PSMI range, presenting a more diffuse validation challenge. In contrast, the sharper, more concentrated peaks at other sites delineate a more acute but spatially confined high-uncertainty zone.

In summary, while the PBE is a universal feature, our analysis demonstrates that it is not a monolithic entity. Its strength (peak rd) and spatial footprint (RS-IRCI) are critically influenced by regional water optical properties. This finding necessitates a context-aware application of the PSMI framework, where validation protocols and uncertainty budgets account for these geographically dependent characteristics of pixel-scale spatial representativeness errors.

### 5.3. Methodological Framework for PSMI Peak Analysis

We introduced the peak concentration index based on the Riemann Stieltjes integral to quantify the spatial concentration of the rd at pixel boundaries. This method draws on concepts from classical indicators, such as the Gini coefficient and Moran’s I, but was particularly developed to address the problem in this study [56,60].

The classic Gini coefficient, as a measure of distribution inequality, focuses on the concentration of the values themselves, while its characterization of the specific location and pattern of high-value clustering in physical space is relatively indirect [56,57]. Similarly, Moran’s I is a powerful tool for detecting spatial autocorrelation, but its analytical effectiveness mainly relies on a predefined spatial adjacency relationship [60,61]. Thus, its applicability may be challenging when analyzing a continuous spatial response field within a pixel that lacks a prior structural definition (see Supplementary Section S3 Figure S3 and Tables S1–S3) [75].

In comparison, the proposed RS-IRCI method aims to respond more directly to the core question of where high deviation values cluster spatially. Its advantage lies in its intrinsic and adaptive nature: it does not require preset spatial weights or external parameters. Instead, it directly utilizes the coordinate and amplitude sequence of the observation data. It directly characterizes the spatial concentration pattern by quantifying the integral relationship between the cumulative cost in spatial extent (Px) for high-deviation samples and their cumulative contribution in amplitude (Py).

This characteristic makes it particularly suitable for characterizing the deviation peak in the PSMI dimension. The sharp morphology presented by the PSMI rd curve is an intuitive manifestation of the spatial clustering of high deviation values within specific pixel boundary intervals (Figure 5 and Figure 6); the RS-IRCI transforms this morphological feature into a quantifiable scalar. A smaller value indicates that high deviations are concentrated in a narrower PSMI range, thereby providing strong numerical evidence for the objective existence of the PBE window. This method not only enables an objective comparison of peak concentration between MODIS and OLCI sensors (Figure 6) but also lays the mathematical foundation for the subsequent precise definition of the PBE window boundaries.

Therefore, the RS-IRCI can be regarded as a supplement to the existing spatial analysis toolkit. It is not intended to replace classical indicators but provides a more targeted solution for specific problems (e.g., spatial high-value concentration), which require fine characterization of the association between spatial location and amplitude [76].

### 5.4. Limitations and Future Work

The constructed PSMI analysis framework provides an innovative approach to analyze the PSE in ocean color remote sensing, yet several aspects of its applicability warrant discussion. As an initial methodological demonstration, this study focused on a representative benchmark site, a choice that offered a controlled environment for the proof-of-concept but necessitates future validation across diverse aquatic environments—such as eutrophic lakes and turbid coastal waters—to establish broader applicability. Our analysis further qualitatively reveals interactions between PBE and other error sources, pointing to the need for future studies to quantitatively dissect their individual contributions (e.g., via variance partitioning). From a methodological standpoint, the framework’s reliance on complete pixel geometry information may currently constrain its application across all sensor types, while its performance is also influenced by the density of in situ validation networks. Additionally, the band-dependent characteristics of the PBE warrant deeper spectral investigation.

Next, we will promote the integration of this method with multisource remote sensing data, especially its application in next-generation ocean color sensors. The pixel spatial effect will be systematically incorporated into the remote sensing product validation system by constructing more refined uncertainty propagation models. These tasks will facilitate the establishment of standardized validation procedures and provide sustained support for enhancing the accuracy and reliability of remote sensing data products.

## 6. Conclusions

This study demonstrates that the Pixel Boundary Effect constitutes a systematic and sensor-dependent source of uncertainty in ocean color validation. Through the proposed Pixel-level Spatial Mismatch Index (PSMI), we observed consistent deviation peaks in R_rs_ when ground sites were located within the pixel’s edge attenuation zone. Specifically, OLCI exhibited peaks closer to the pixel interior (PSMI: −0.097 to −0.035 km) with magnitudes up to 546.3% at 412 nm, while MODIS showed broader spatial distribution (PSMI: −0.166 to 0.126 km). The exclusion of PBE-influenced samples improved the MODIS validation R^2^ at 490 nm from 0.662 to 0.972, indicating that PBE contributes substantially to high deviation outliers.

To objectively quantify this effect, the Riemann-Stieltjes Integral-based Range Concentration Index (RS-IRCI) was introduced, which confirmed spatial clustering of deviations with values below 0.43. Combined with the PBE window derived from Cumulative Normalized Standard Deviation (CNSD), this framework offers an objective basis for identifying boundary-affected samples and supports future automated quality screening.

Error source migration further revealed that PBE acts both independently and interactively. Up to 20.6% (MODIS, 490 nm) of samples were reclassified as PBE-induced or PBE-related multi-factor errors following its incorporation, underscoring its role in uncertainty budgets. These results establish PBE as a quantifiable error source and suggest that the integration of pixel-scale metrics such as PSMI and RS-IRCI may enhance the robustness of future validation protocols.

## Figures and Tables

**Table 1:** AERONET-OC station information.

Name	Longitude (°)	Latitude (°)	Height (m)	Sample Size	
				MODIS	OLCI
AAOT	12.508	45.314	10	8760	4284
Casablanca_Platform	1.358	40.717	35	5100	3336
Galata_Platform	28.193	43.045	31	9480	4680

**Table 2:** Error Source Flags of Level-2 Ocean Color Products in MODIS and OLCI.

Flag	Explanation	Sensor	Categories	Feature	
				Independent	Interactive
LOWLW	Low Water-Leaving Radiance	MODIS, OLCI	Atmosphere and Surface	√	√
COCCOLITH	Coccolithophore Bloom	MODIS, OLCI		√	√
TURBIDW	Turbid Water	MODIS, OLCI		√	√
MODGLINT	Moderate Sun Glint	MODIS, OLCI		√	√
HIGLINT	High Sun Glint	OLCI			√
HIPOL	High Polarization	MODIS		√	√
HISOLZEN	High Solar Zenith Angle	MODIS, OLCI	Observation	√	√
HISATZEN	High Satellite Zenith Angle	MODIS		√	√
STRAYLIGHT	Stray Light	MODIS, OLCI	Sensors	√	√
ATMWARN	Atmospheric Warning	MODIS, OLCI	Model and Algorithm		√
MAXAERITER	Aerosol Iteration Maximum Reached	MODIS, OLCI			√

**Table 3:** Validation metrics for samples within the PBL.

Satellite Products	MODISA_L2_OC			OLCIS3A_L2_EFR_OC		
Band (nm)	R^2^ (*p*-Value < 0.05)	AVG (%)	STD (%)	R^2^ (*p*-Value < 0.05)	AVG (%)	STD (%)
412	0.820	23.966	17.846	0.747	32.920	25.154
443	0.910	16.897	12.437	0.850	29.267	19.513
490	0.972	8.144	5.248	0.865	23.762	17.369
667	0.653	36.913	24.285	0.829	35.610	23.990

**Table 4:** PBE information of AAOT.

Satellite Products	MODISA_L2_OC			OLCIS3A_L2_EFR_OC		
Band (nm)	Loc. of Peak (km)	Peak Value (%)	RS-IRCI	Loc. of Peak (km)	Peak Value (%)	RS-IRCI
412	0.057	277.589	0.433	−0.035	501.329	0.507
443	0.057	201.272	0.474	−0.035	413.497	0.502
490	0.022	129.924	0.460	−0.035	236.958	0.529
667	−0.335	310.902	0.415	−0.076	304.538	0.611

**Table 5:** PBE information of Casablanca platform.

Satellite Products	MODISA_L2_OC			OLCIS3A_L2_EFR_OC		
Band (nm)	Loc. of Peak (km)	Peak Value (%)	RS-IRCI	Loc. of Peak (km)	Peak Value (%)	RS-IRCI
412	−0.094	90.085	0.418	−0.017	73.359	0.487
443	0.126	62.097	0.478	−0.073	81.170	0.411
490	0.022	55.550	0.383	−0.073	60.729	0.391
667	0.022	507.352	0.197	−0.097	1038.695	0.298

**Table 6:** PBE information of Galata platform.

Satellite Products	MODISA_L2_OC			OLCIS3A_L2_EFR_OC		
Band (nm)	Loc. of Peak (km)	Peak Value (%)	RS-IRCI	Loc. of Peak (km)	Peak Value (%)	RS-IRCI
412	−0.091	376.157	0.337	−0.059	546.261	0.289
443	−0.166	227.971	0.398	−0.032	305.152	0.411
490	−0.166	214.956	0.385	−0.032	150.010	0.418
667	−0.299	354.986	0.430	−0.032	657.401	0.252

## Data Availability

The data presented in this study are available upon request from the authors.

## Supplementary Materials

The following supporting information can be downloaded at: https://www.mdpi.com/article/10.3390/s25237333/s1, Figure S1: Definition of pixel-level spatial variables; Figure S2: Evaluation of pixel reconstruction and PSMI algorithms against the reference corner point product (OMPIXCOR); Figure S3: Typical peak distributions and remote sensing datasets; Table S1: Model expressions and parameters; Table S2: Peak concentration quantification metrics; Table S3: Calculated values of peak concentration metrics.
