# Supplementary material for: Bridging Gaps in Aquatic Remote Sensing Reflectance Validation: Pixel Boundary Effect and Its Induced Errors
Source: Sensors (Basel). 2025 Dec 2;25(23):7333. doi: 10.3390/s25237333 (PMC12694536; doi:10.3390/s25237333)
Supplement: Supplementary file 1 [file sensors-25-07333-s001.zip › sensors-3956469-supplementary.pdf]

Supplementary

Bridging Gaps in Aquatic Remote Sensing Reflectance Validation: Pixel Boundary Effect and Its Induced Errors

Shuling Xiao [1,†], Chunguang Lyu [1,*,†], Chi Zhang [1,2], Jochem Verrelst [3], Ling Wang [4], Yunfei Shi [1], Yanmei Lyu [1], Haochuan Shi [1]

[1] College of Resources and Environment, Linyi University, Linyi 276000, China; 202018090131@lyu.edu.cn (S.X.); zc@ms.xjb.ac.cn (C.Z.); shiyunfei@lyu.edu.cn (Y.S.); lvyanmei@lyu.edu.cn (Y.L.); 250857042204@lyu.edu.cn (H.S.)
[2] State Key Laboratory of Desert and Oasis Ecology, Xinjiang Institute of Ecology and Geography, Chinese Academy of Sciences, Urumqi 830011, China
[3] Image Processing Laboratory (IPL), University of Valencia, Valencia 46980, Spain; jochem.verrelst@uv.es
[4] Key Laboratory of Radiometric Calibration and Validation for Environmental Satellites, China Meteorological Administration, Beijing 100081, China; lingw@cma.cn
* Correspondence: lvchunguang@lyu.edu.cn
† These authors contributed equally to this work.

Section S1

This section presents the variables involved in Equations (3)–(7). As shown in **Figure S1**, the schematic diagram graphically illustrates the spatial configuration and interrelationships of the key geometric variables. It provides an intuitive and rigorous visual representation that greatly facilitates the understanding of the PSMI derivation process and its underlying physical principles.

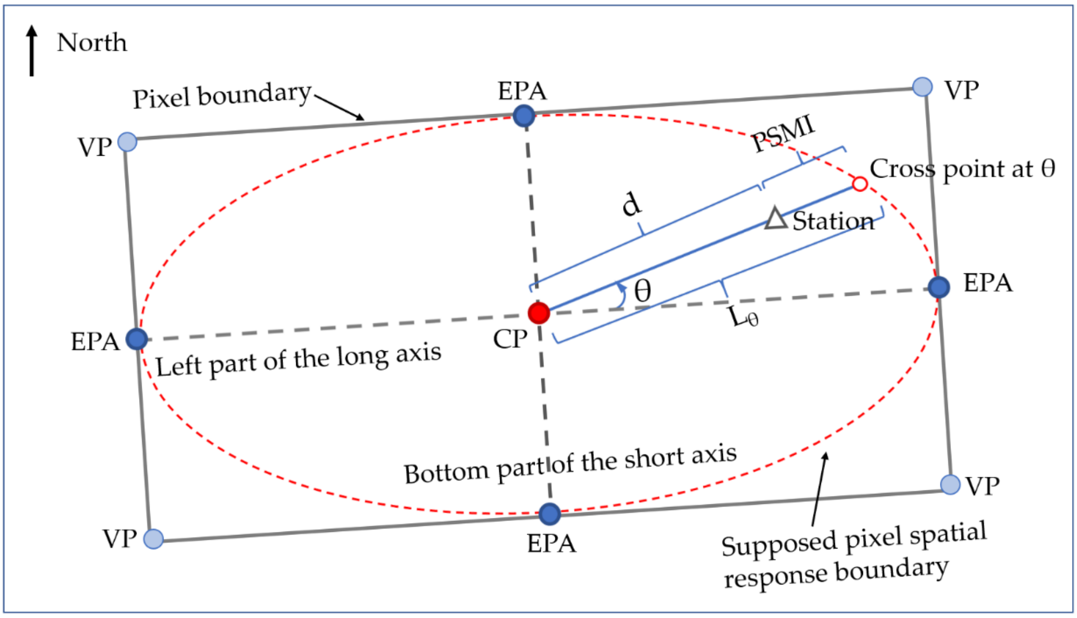

Figure S1 Definition of pixel-level spatial variables

Section S2

To quantify the accuracy of our pixel reconstruction and PSMI algorithm, we performed a direct comparison with the authoritative OMI Pixel Corner (OMPIXCOR) product [1]. We randomly selected a site near our study area, the Academy of Athens (23.78°E, 37.99°N), and applied our method to 502 OMI overpass samples from 2020, deriving key parameters: pixel corner coordinates, long/short axes, and the PSMI. As shown in Figure S2, our algorithm demonstrates excellent agreement with the OMPIXCOR reference data. The derived pixel corner longitudes and latitudes exhibit high correlation ($R^2 > 0.985$) with RMSEs of less than 0.026°. The lengths of the major and minor axes, which are critical for defining the elliptical model used in PSMI calculation [2], also show strong consistency, aligning with known OMI pixel characteristics [3]. Consequently, the final PSMI values are highly reproducible. This validation confirms that our method robustly reconstructs pixel geometry against the trusted benchmark, ensuring the reliability of the PSMI for spatial representativeness analysis.

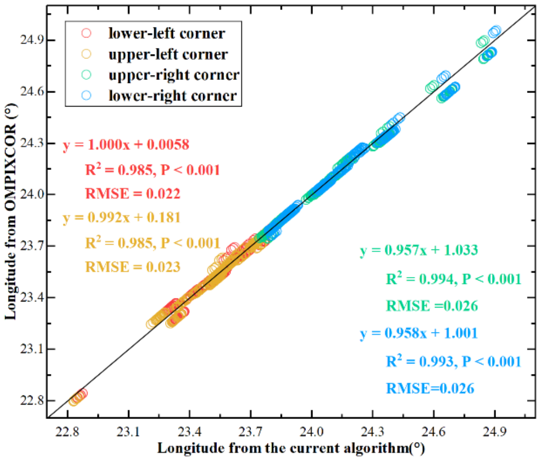

(a) Longitude of corner point

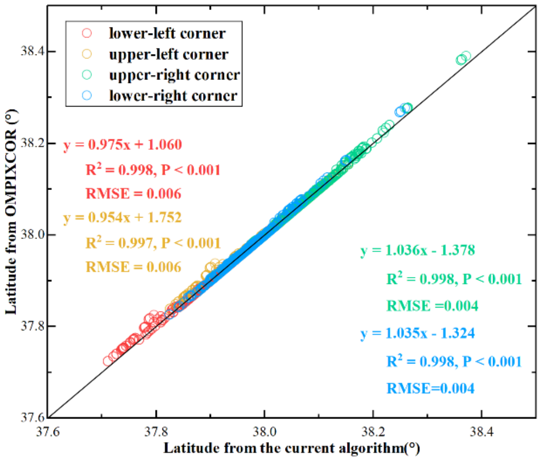

(b) Latitude of corner point

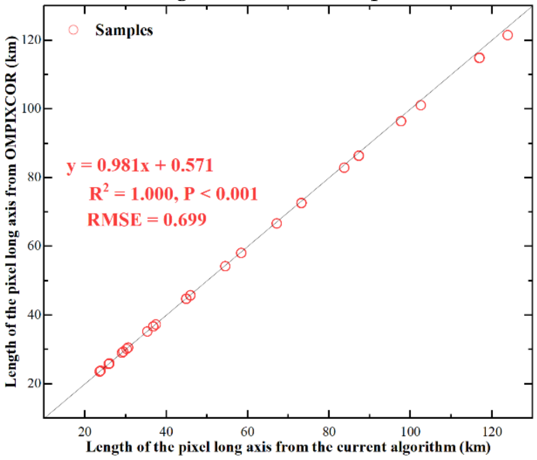

(c) Length of the pixel long axis

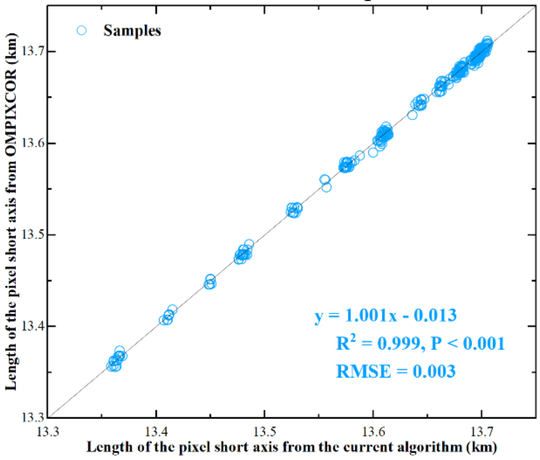

(d) Length of the pixel short axis

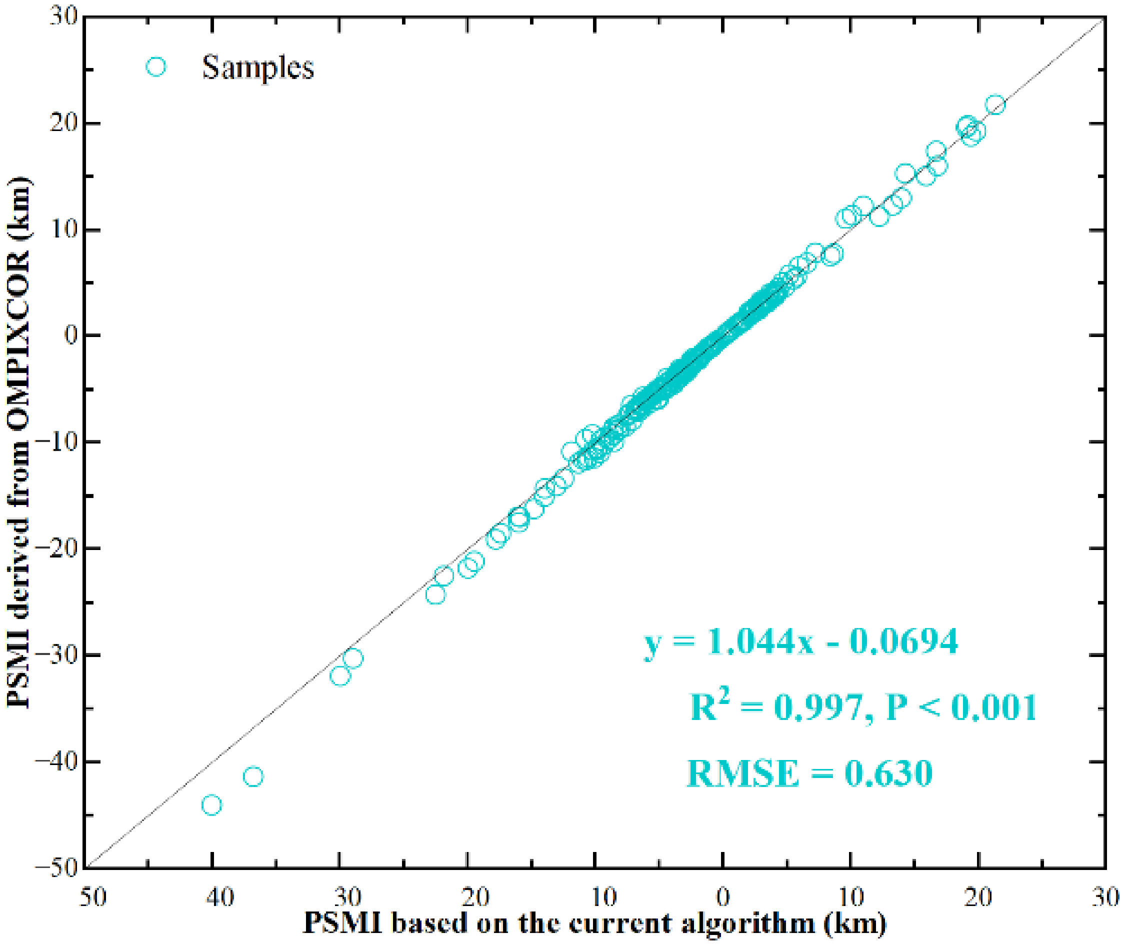

(e) PSMI

Figure S2 Figure S2 Evaluation of pixel reconstruction and PSMI algorithms against the reference corner point product (OMPIXCOR)

Section S3

This section provides supporting information on the interpretation and validation of the RS-IRCI metric referenced in the main text. **Figure S3** illustrates several typical peak distribution patterns, including analytical models and a real remote sensing PSMI profile. **Table S** summarizes the mathematical expressions and parameters for the analytical models shown in **Figure S3**. **Table S** defines the formulas of several comparative metrics used for benchmarking, and **Table S** presents the calculated values of these metrics for each distribution, including the proposed RS-IRCI.

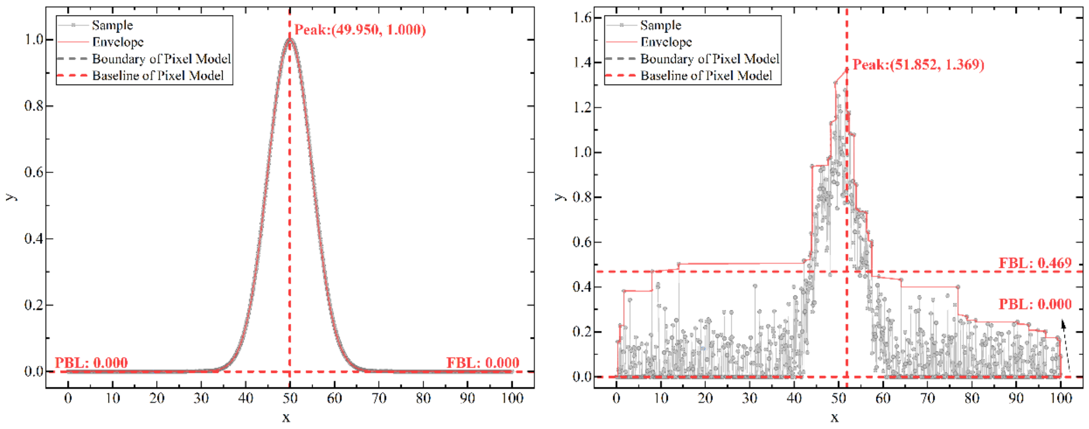

(a) Gaussian single Peak Distribution (b) Noisy Gaussian Single Peak Distribution (ϵ = 0.15)

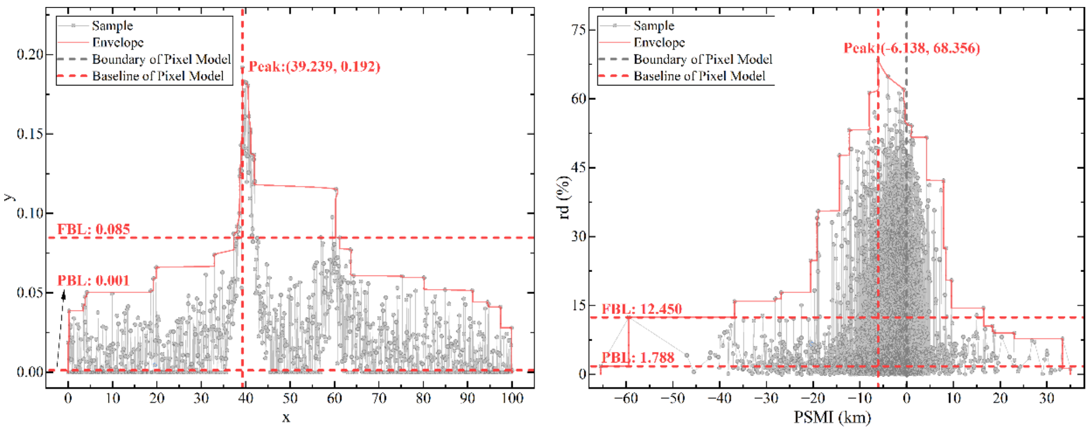

(c) Noisy Cauchy Mixture Distribution (ϵ = 0.024) (d) OMDOAO3 PSMI curve (2020 ~ 2022) [1]

Figure S3 Typical peak distributions and remote sensing datasets

Table S1 Model expressions and parameters [4,5]

Name	Expression	Parameter
Gaussian single Peak Distribution (y)	$y = A \cdot e^{-0.5\left(\frac{x-\mu}{\sigma}\right)^2}$	μ: 50; A: 1; σ: 5
Cauchy Mixture Distribution (y)	$y = \sum_{i=1}^{2} A_i \cdot \frac{1}{\pi\gamma_i\left[1+\left(\frac{x-\mu_i}{\gamma_i}\right)^2\right]}$	μ: [40, 60]; A: [1, 0.6] 1; γ: [2, 3]
Noise level (ϵ)	$\epsilon \backsim \mathcal{N}(0, \sigma_{noise}^2)$ $y_{noisy}(x) = y_{original}(x) + \epsilon$	$\mathcal{N}$: Normal distribution; σ_{noise}^2:Variance of noise

Table S2 Peak concentration quantification metrics [6-11]

Name	Expression	Condition
Standard Gini Coefficient (G)	$G = \frac{\sum_{i=1}^{n}(2i-n-1) \cdot y_i}{n \cdot \sum_{i=1}^{n} y_i}$	For the sorted sequence: $y_1 \leq y_2 \leq \ldots \leq y_3$
Position-weighted Gini Coefficient (G_p)	$G_p = \frac{\sum_{i=1}^{n}\sum_{j=1}^{n} y_i \cdot y_j \cdot \lvert x_i - x_j \rvert}{2 \cdot (x_{max} - x_{min}) \cdot (\sum_{i=1}^{n} y_i)^2}$	For the sequence sorted by position: $(x_1, y_1) \leq (x_2, y_2) \leq \ldots \leq (x_n, y_n)$, where $x_1 \leq x_2 \leq \ldots \leq x_3$

Weighted Standard Deviation (σ_w)	$\sigma_w = \sqrt{\frac{\sum_{i=1}^{n} w_i \cdot (x_i - \overline{x}_w)^2}{\sum_{i=1}^{n} w_i}}$<br>$\overline{x}_w = \frac{\sum_{i=1}^{n} w_i \cdot x_i}{\sum_{i=1}^{n} w_i}$	x: Position coordinate<br>w: Weight (usually the height value y)
Moran's I for one-dimensional sequence (I)	$I = \frac{\sum_{i=1}^{n} \sum_{j=1}^{n} w_{ij} (y_i - \overline{y})(y_j - \overline{y})}{\sum_{i=1}^{N} (y_i - \overline{y})^2}$	w_{ij}: 1/k, if d_{ij} ranks among the k smallest in $\{d_{i1}, d_{i2}, \ldots, d_{iN}\}$; otherwise, 0<br>$d_{ij} = \lvert x_i - x_j \rvert$

Table S3 Calculated values of peak concentration metrics

Evaluation Metrics	Reference Value	Gaussian Single Peak Distribution	Noisy Gaussian Single Peak Distribution	Noisy Cauchy Mixture Distribution	OMDOAO3 PSMI curve (2020 ~ 2022)
G	[0, 1]	0.841	0.721	0.659	0.531
G_p	[0, 1]	0.000	0.002	0.000	0.243
σ_w	[0, +∞)	0.05	0.173	0.203	0.051
I	[-1, 1]	N/A	0.876	0.763	0.202
η (RS-IRCI)	[0, 1]	0.125	0.348	0.374	0.331

Reference

1. Kurosu, T.P.; Celarier, E.A. OMI/Aura Global Ground Pixel Corners 1-Orbit L2 Swath 13x24km V003. NASA Goddard Earth Sciences Data and Information Services Center (GES DISC), 2010. doi:10.5067/Aura/OMI/DATA2020.
2. Lyu, C.; Zhang, W.; Zhang, C.; Shi, Y.; Zhang, Y.; Wang, Y. Evaluating the Spatial Representativeness of Ground-Based Observations for Satellite Total Ozone Products. *International Journal of Applied Earth Observation and Geoinformation* **2024**, *129*, 103778, doi:10.1016/j.jag.2024.103778.
3. de Graaf, M.; Sihler, H.; Tilstra, L.G.; Stammes, P. How Big Is an OMI Pixel? *Atmospheric Measurement Techniques* **2016**, *9*, 3607–3618, doi:10.5194/amt-9-3607-2016.
4. Bryc, W. Normal Distributions. In *The Normal Distribution: Characterizations with Applications*; Springer, 1995; pp. 23–38.
5. Kahrari, F.; Rezaei, M.; Yousefzadeh, F.; Arellano-Valle, R.B. On the Multivariate Skew-Normal-Cauchy Distribution. *Statistics & Probability Letters* **2016**, *117*, 80–88, doi:10.1016/j.spl.2016.05.005.
6. Zheng, X.; Xia, T.; Yang, X.; Yuan, T.; Hu, Y. The Land Gini Coefficient and Its Application for Land Use Structure Analysis in China. *PLoS ONE* **2013**, *8*, e76165, doi:10.1371/journal.pone.0076165
7. Zhou, T.; Ni, C.; Zhang, M.; Xia, P. Assessing Spatial and Temporal Distribution of Algal Blooms Using Gini Coefficient and Lorenz Asymmetry Coefficient. *Front. Environ. Sci.* **2022**, *10*, 810902, doi:10.3389/fenvs.2022.810902.
8. Yuan, Q.; McIntyre, N.; Wu, Y.; Liu, Y.; Liu, Y. Towards Greater Socio-Economic Equality in Allocation of Wastewater Discharge Permits in China Based on the Weighted Gini Coefficient. *Resources, Conservation and Recycling* **2017**, *127*, 196–205, doi:10.1016/j.resconrec.2017.08.023.
9. Xie, J.; Liu, X.; Wang, M. SFKNN-DPC: Standard Deviation Weighted Distance Based Density Peak Clustering Algorithm. *Information Sciences* **2024**, *653*, 119788, doi:10.1016/j.ins.2023.119788.
10. Zhang, C.; Lv, W.; Liu, G.; Wang, Y. Multidimensional Spatiotemporal Autocorrelation Analysis Theory Based on Multi-Observation Spatiotemporal Moran's I and Its Application in Resource Allocation. *Earth Sci Inform* **2025**, *18*, 36, doi:10.1007/s12145-024-01598-8.
11. Song, Y.; Song, J. Analysis of Surface Temperature in an Urban Area Using Supervised Spatial Autocorrelation and Moran's I. *Earth Sci Inform* **2022**, *15*, 2545–2552, doi:10.1007/s12145-022-00856-x.
